# Supplementary figures and images for: The value of citizen science for ecological monitoring of mammals
Source: PeerJ. 2018 Mar 29;6:e4536. doi: 10.7717/peerj.4536 (PMC5878931; doi:10.7717/peerj.4536)

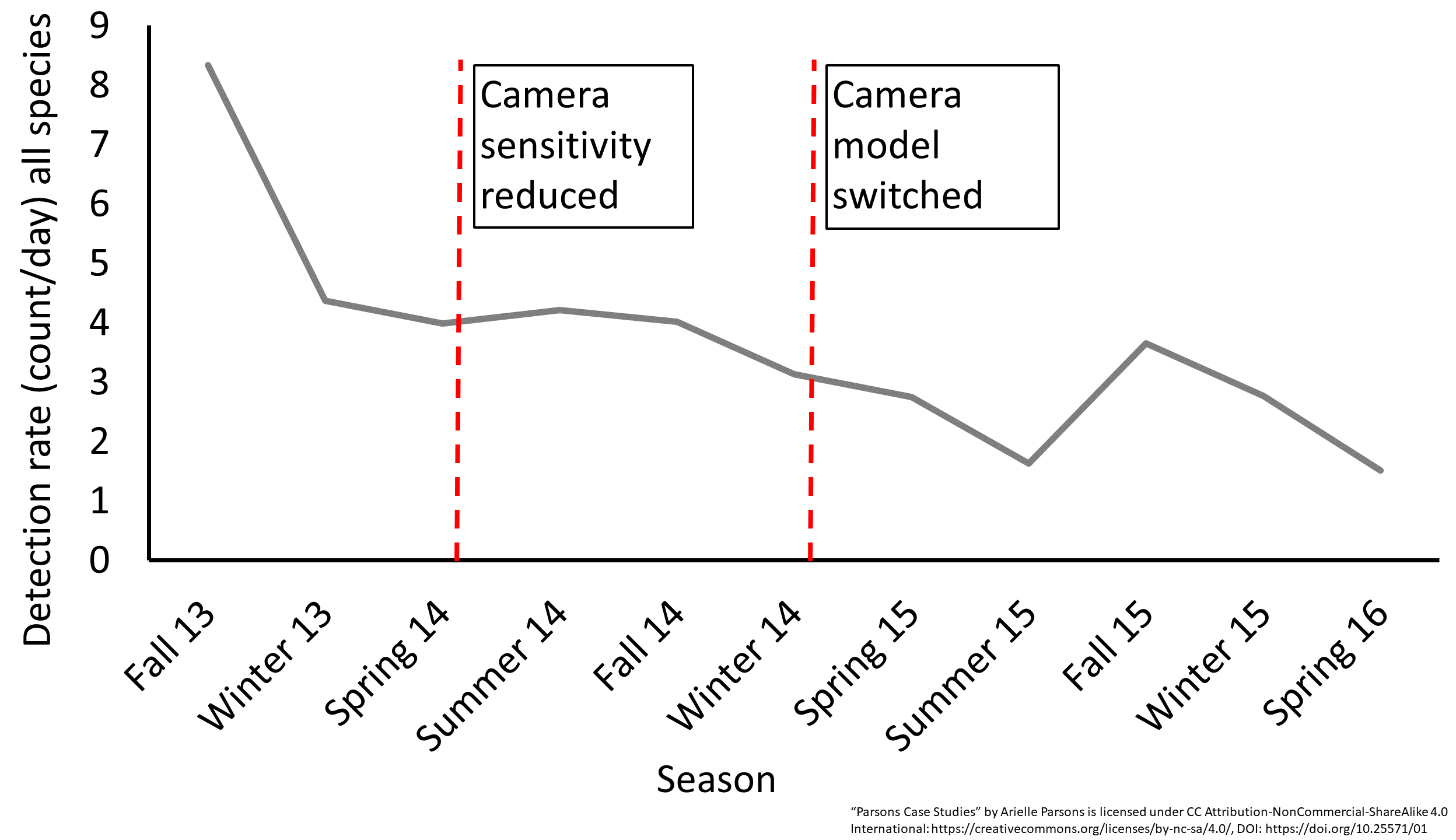

Supplement: Figure S1 — Data were taken from camera traps run at Prairie Ridge Ecostation, Raleigh, NC, USA between 2013 and 2016. [file peerj-06-4536-s001.png]

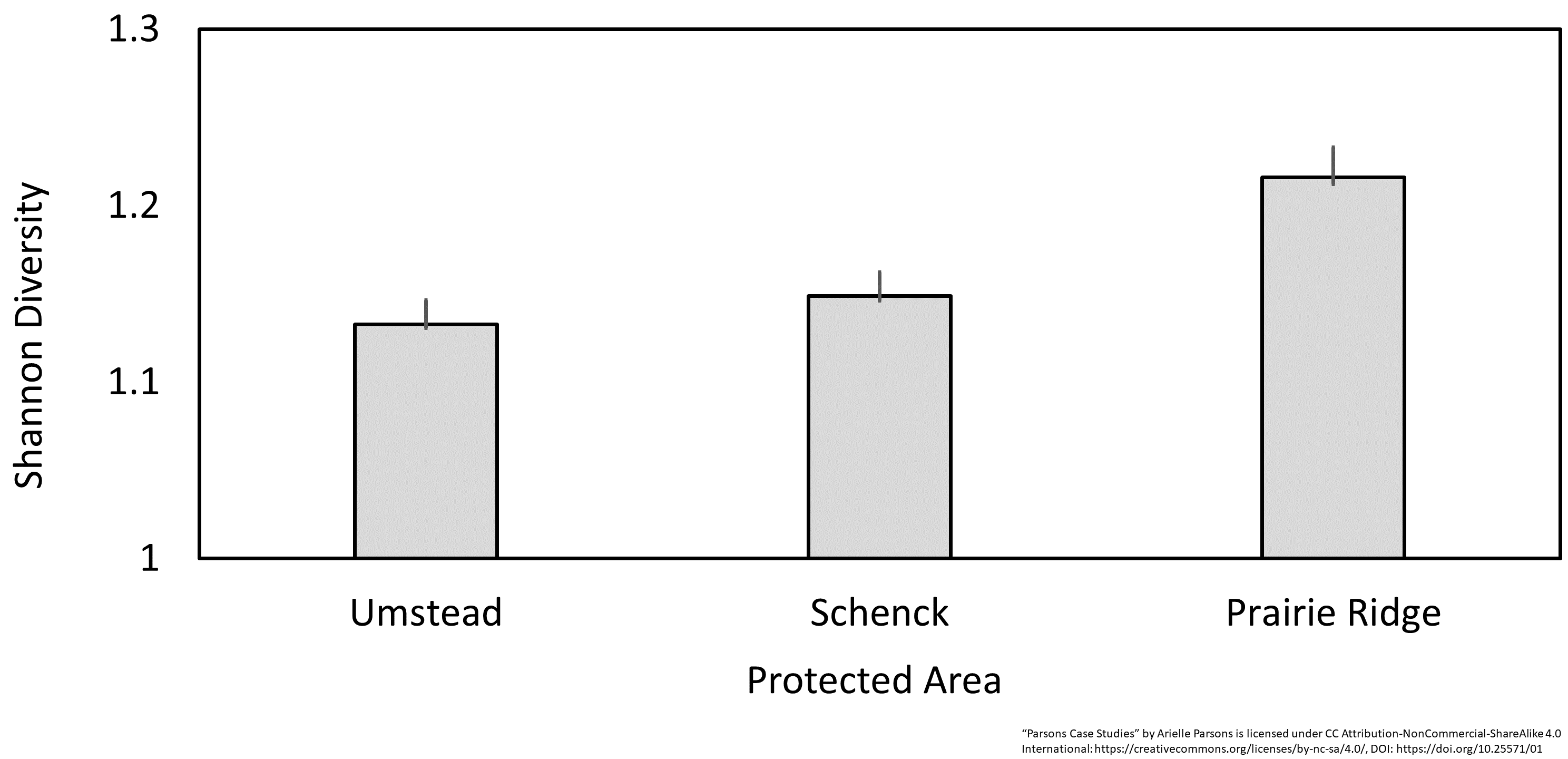

Supplement: Figure S2 — Data were taken from camera traps run in Fall 2013. [file peerj-06-4536-s002.png]

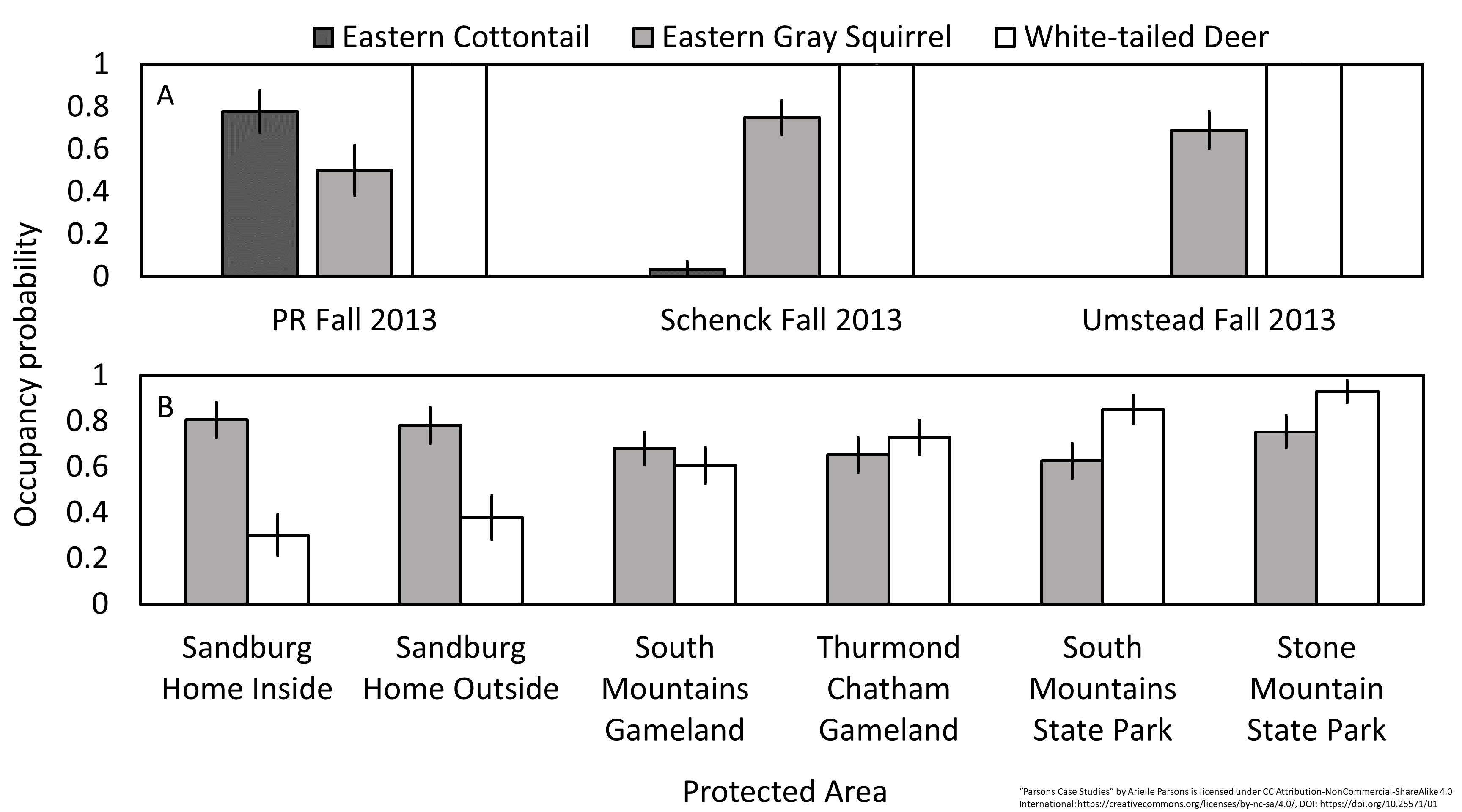

Supplement: Figure S3 — Prairie Ridge Ecostation (A) was sampled in Fall 2013 and compared to two nearby sites sampled during the same timeframe and Carl Sandburg Home National Historic Site (B) was sampled in Fall 2015 and compared to four nearby sites sampled in Fall 2012. [file peerj-06-4536-s003.png]

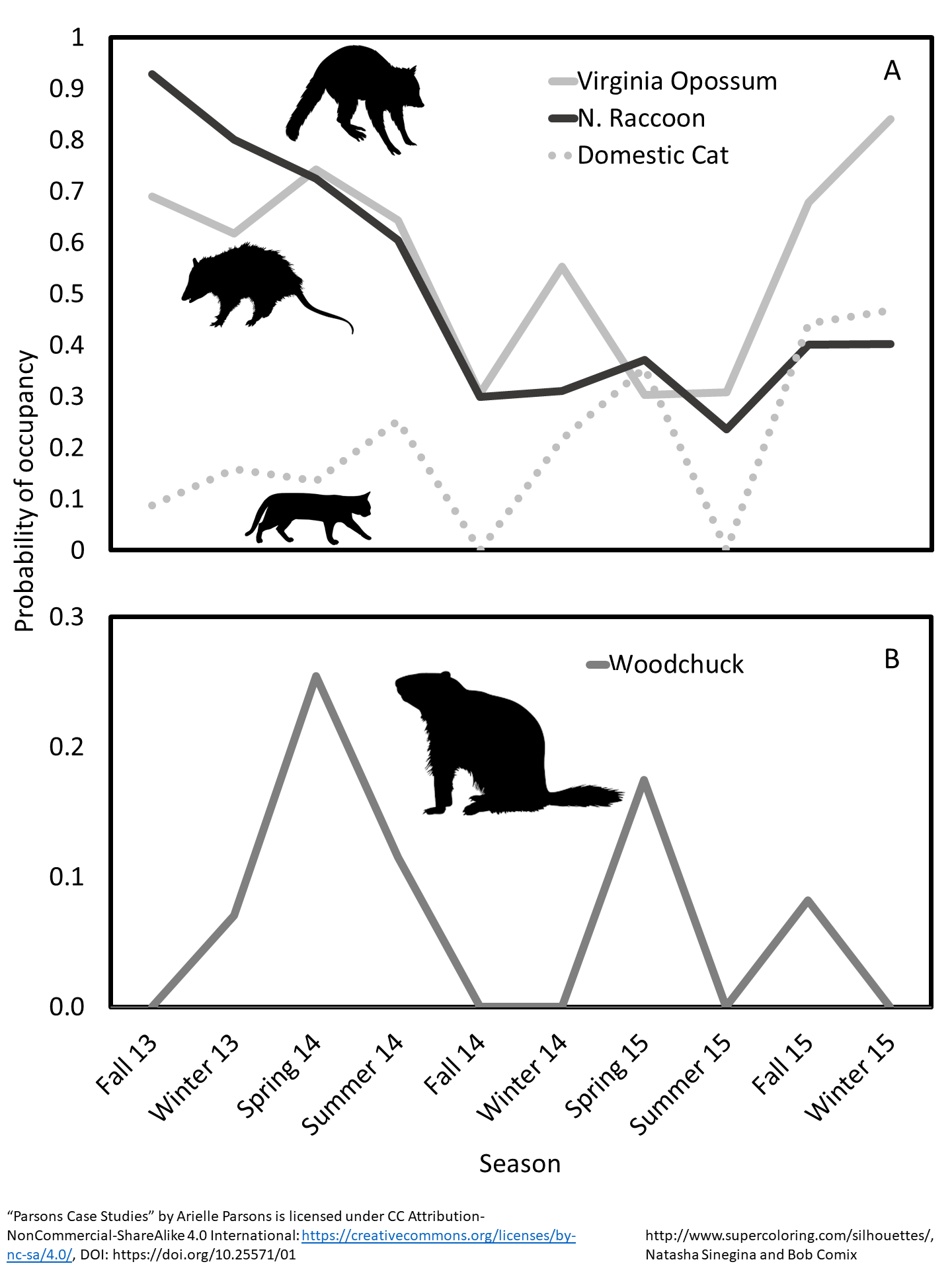

Supplement: Figure S4 — Data were taken from camera traps run between 2013 and 2016. [file peerj-06-4536-s004.png]

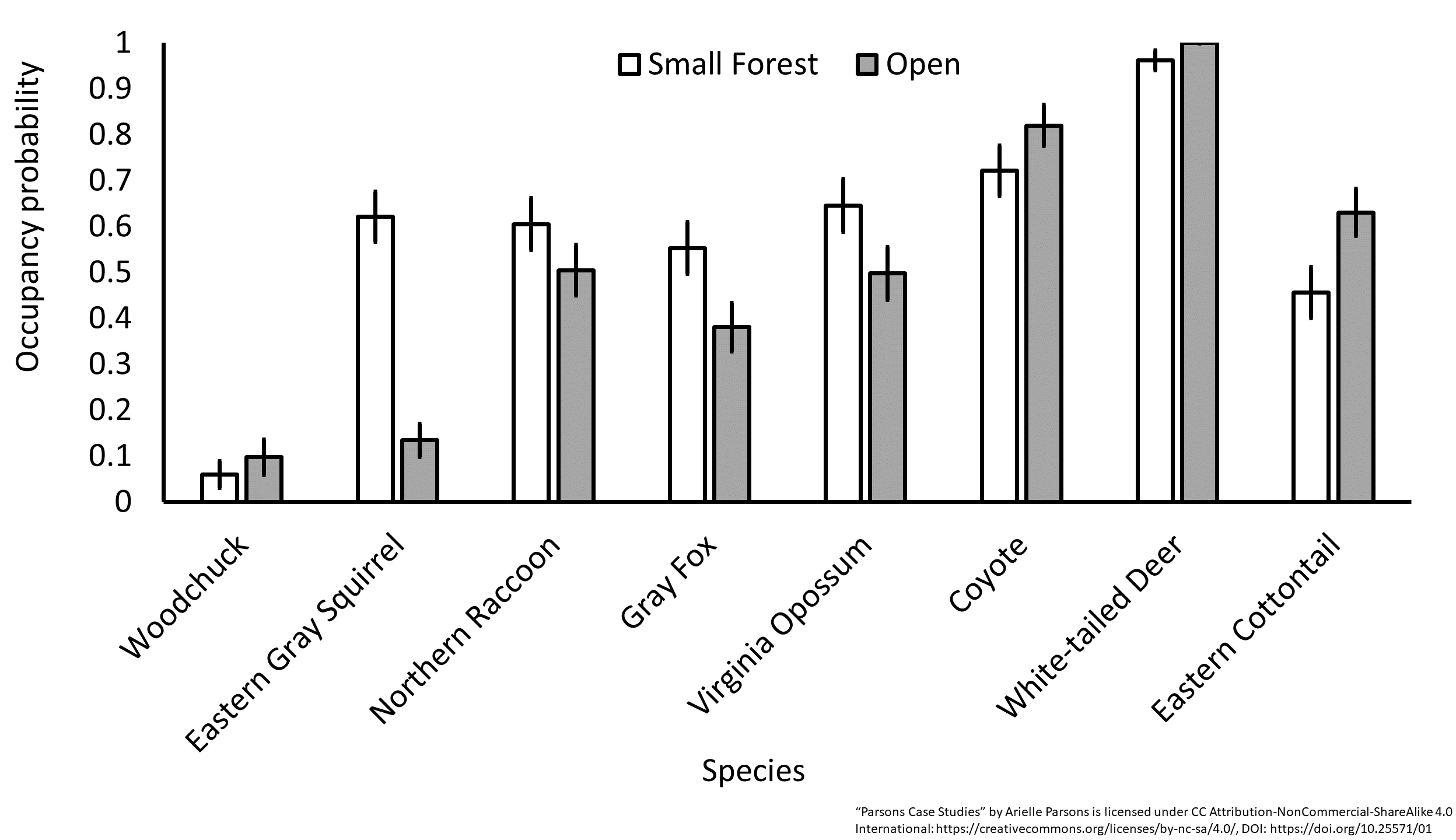

Supplement: Figure S5 — Data were taken from camera traps run from 2013 to 2016. [file peerj-06-4536-s005.png]

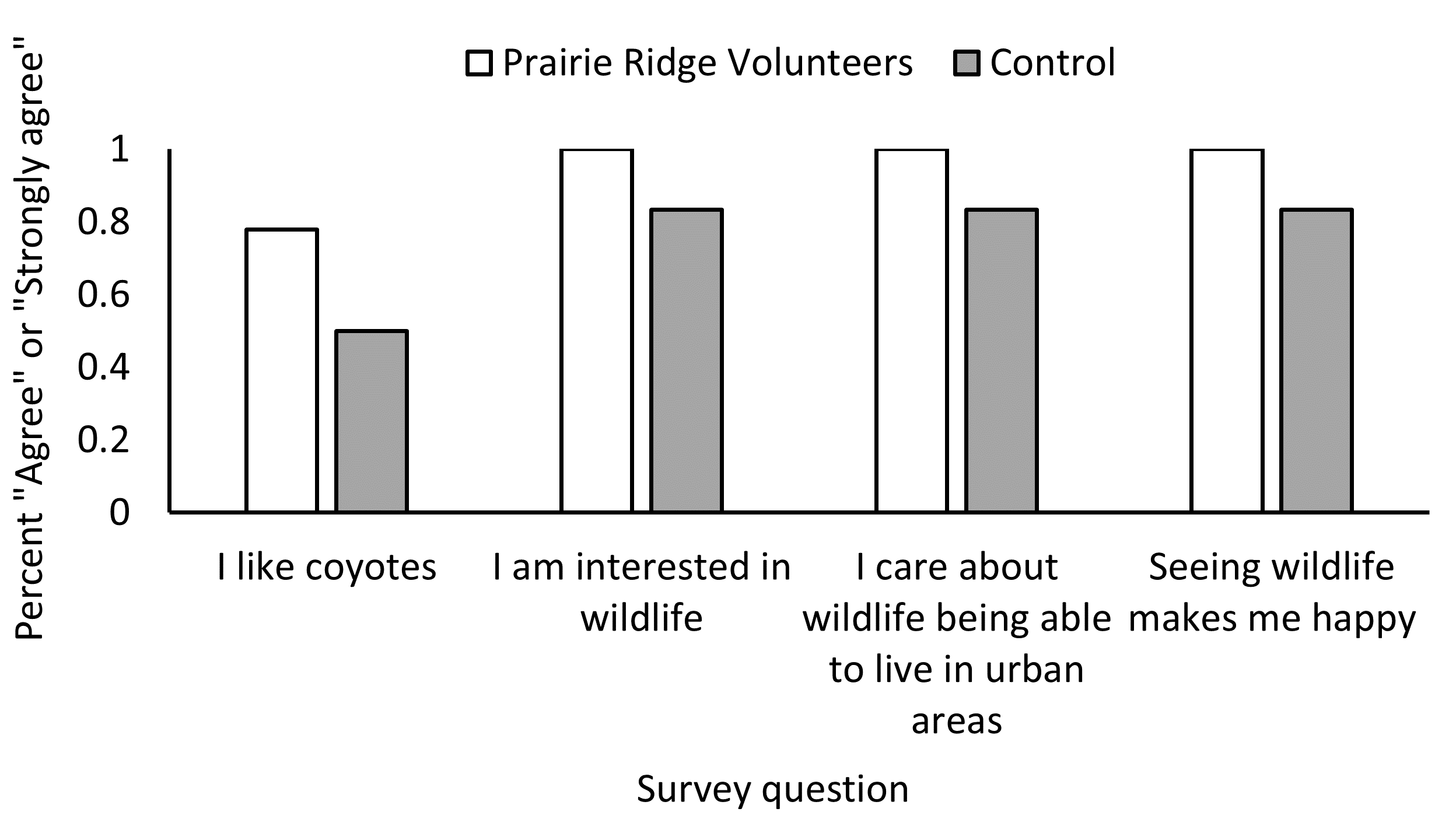

Supplement: Figure S6 — Results show the percent of respondents in two groups (Prairie Ridge volunteers and control) that agreed or strongly agreed with each statement. [file peerj-06-4536-s006.png]
