# Supplementary material for: The value of citizen science for ecological monitoring of mammals
Source: PeerJ. 2018 Mar 29;6:e4536. doi: 10.7717/peerj.4536 (PMC5878931; doi:10.7717/peerj.4536)
Supplement: Table S1 — The two groups are: control group (A) and Prairie Ridge participants (B). [file peerj-06-4536-s007.docx]

Table S1 : Survey questions for control group (A) and Prairie Ridge participants (B)

1. Control group survey questions

*Choose the response that best describes how you feel about each sentence*

1.  I like coyotes

1. strongly agree b. agree   c. neutral   d. disagree  e. strongly disagree

2.  I am very interested in wildlife

a. strongly agree    b. agree    c. neutral  d. disagree  e. strongly disagree

3.  I do not care about wildlife being able to live in urban areas

a. strongly agree    b. agree  c. neutral d. disagree    e. strongly disagree

4.  I have never had a negative experience with wildlife in urban areas

a. strongly agree    b. agree  c. neutral d. disagree    e. strongly disagree

5.  Seeing wildlife makes me happy

a. strongly agree    b. agree  c. neutral d. disagree    e. strongly disagree

6. Have you ever participated in citizen science?

a. No    b. Yes

7.  I would be interested in participating in citizen science at an urban nature center

a. strongly agree    b. agree  c. neutral d. disagree    e. strongly disagree

1. Prairie Ridge participants survey questions

*Choose the response that best describes how you feel about each sentence*

1.  I did not like coyotes **before** working with camera traps at Prairie Ridge.

1. strongly agree b. agree   c. neutral   d. disagree  e. strongly disagree

2.  I did not like coyotes any more **after** working with camera traps at Prairie Ridge than before I worked there.

a.   strongly agree    b. agree   c. neutral   d. disagree   e. strongly disagree

3.  I was very interested in wildlife **before** participating in camera trapping at Prairie Ridge.

a. strongly agree    b. agree    c. neutral  d. disagree  e. strongly disagree

4.  I am very interested in wildlife NOW.

1. strongly agree    b. agree   c. neutral   d. disagree   e. strongly disagree

3.  I do not care about wildlife being able to live in Raleigh

a. strongly agree    b. agree  c. neutral d. disagree    e. strongly disagree

4.  Seeing wildlife makes me happy.

a. strongly agree    b. agree  c. neutral d. disagree    e. strongly disagree

5. Participating in the camera trapping project at Prairie Ridge has made me feel more comfortable being in the environment and talking with the researchers at Prairie Ridge

a. strongly agree    b. agree  c. neutral d. disagree    e. strongly disagree

6. Did you become interested in any of the animals?

7.  Did you get anything out of the experience that you liked?

8.  Did you have any experiences that you didn’t like?
